# Expected Anomalies in the Fossil Record

**DOI:** 10.4137/ebo.s555

**Published:** 2008-03-18

**Authors:** Mareike Fischer, Mike Steel

**Affiliations:** Allan Wilson Centre for Molecular Ecology and Evolution, Biomathematics Research Centre, University of Canterbury, Private Bag 4800, Christchurch, New Zealand

**Keywords:** fossil record, null models, phylogenetic trees

## Abstract

The problem of intermediates in the fossil record has been frequently discussed ever since Darwin. The extent of ‘gaps’ (missing transitional stages) has been used to argue against gradual evolution from a common ancestor. Traditionally, gaps have often been explained by the improbability of fossilization and the discontinuous selection of found fossils. Here we take an analytical approach and demonstrate why, under certain sampling conditions, we may not expect intermediates to be found. Using a simple null model, we show mathematically that the question of whether a taxon sampled from some time in the past is likely to be morphologically intermediate to other samples (dated earlier and later) depends on the shape and dimensions of the underlying phylogenetic tree that connects the taxa, and the times from which the fossils are sampled.

## Introduction

Since Darwin’s book *On the Origin of Species by Means of Natural Selection, or the Preservation of Favoured Races in the Struggle for Life* [2], there has been much debate about the evidence for continuous evolution from a universal common ancestor. Initially, Darwin only assumed the relatedness of the majority of species, not of all of them; later, however, he came to the view that because of the similarities of all existing species, there could only be one ‘root’ and one ‘tree of life’ (*cf*. [11]). All species are descended from this common ancestor and indications for their gradual evolution have been sought in the fossil record ever since. Usually, the improbability of fossilization or of finding existing fossils was put forward as the standard answer to the question of why there are so many ‘gaps’ in the fossil record. Such gaps have become popularly referred to as ‘missing links’, i.e. missing intermediates between taxa existing either today or as fossils.

Of course, the existence of gaps is in some sense inevitable: every new link gives rise to two new gaps, since evolution is generally a continuous process whereas fossil discovery will always remain discontinuous. Moreover, a patchy fossil record is not necessarily evidence against evolution from a common ancestor through a continuous series of intermediates—indeed, in a recent approach, Elliott Sober (*cf*. [11]) applied simple probabilistic arguments to conclude that the existence of some intermediates provides a stronger support for evolution than the non-existence of any (or some) intermediates could ever provide for a hypothesis of separate ancestry. Moreover, some lineages appear to be densely sampled, whereas of others only few fossiliferous horizons are known (*cf*. [10]). This problem has been well investigated and statistical models have been developed to master it (see e.g. [6], [7]), [12]).

In this paper, we suggest a further argument that may help explain missing links in the fossil record. Suppose that three fossils can be dated back to three different times. Can we really expect that a fossil from the intermediate time will appear (morphologically) to be an ‘intermediate’ of the other two fossils? We will explore this question via a simple stochastic model.

In order to develop this model, we first state some assumptions we will make throughout this paper: firstly, we will consider that we are sampling fossil taxa of closely related organisms and which differ in a number of morphological characteristics. We assume this group of taxa has evolved in a ‘tree-like’ fashion from some common ancestor; that is, there is an underlying phylogenetic tree, and the taxa are sampled from points on the branches of this tree.

It is also necessary to say how morphological divergence might be related to time, as this is important for deciding whether a taxon is an intermediate or not. In this paper, we make the simplifying assumption that, within the limited group of taxa under consideration (and over the limited time period being considered), the expected degree of morphological divergence between two taxa is proportional to the total amount of evolutionary history separating those two taxa. This evolutionary history is simply the time obtained by adding together the two time periods from the most recent common ancestor of the two taxa until the times from which each was sampled (in the case where one taxon is ancestral to the other, this is simply the time between the two samples). This assumption on morphological diversity would be valid (in expectation) if we view morphological distance as being proportional to the number of discrete characters that two species differ on, provided that two conditions hold: (i) each character has a constant rate of character state change (substitution) over the time frame *T* that the fossils are sampled from, and (ii) *T* is short enough that the probability of a reverse or convergent change at any given character is low. We require these conditions to hold in the proofs of the following results. We will discuss other possible relations of morphological diversification and distance towards the end of this paper.

**Figure 1:**
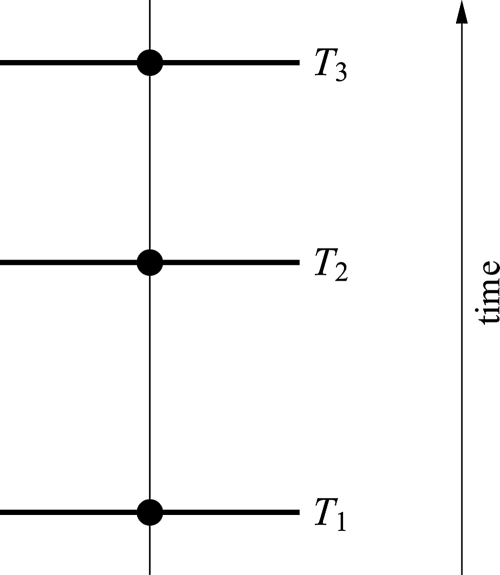
When the tree consists of only one lineage from which samples are taken at times *T*_1_, *T*_2_ and *T*_3_, then clearly the distance *d*_1,3_ is always larger than *d*_1,2_ and *d*_2,3_. Consequently, *E*_1,3_ > max{*E*_1,2_, *E*_2,3_}.

**Figure 2:**
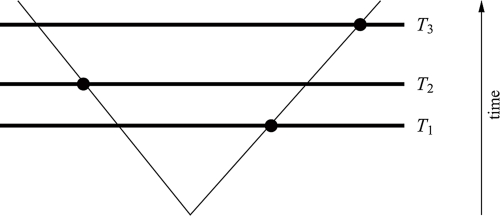
For samples taken from different lineages of a tree, the distance *d*_1,3_ of one particular sample from time *T*_1_ to the one of *T*_3_ can be smaller than the distance of either of them to the sample taken at time *T*_2_. Yet in expectation we always have *E*_1,3_ > max{*E*_1,2_, *E*_2,3_} for two-branch trees. For more complex trees this can fail as we show in Example 2.7.

The simplest scenario is the case where the three samples all lie on the same lineage, so that the evolutionary tree can be regarded as a path (*cf*. Fig. 1). In this case, the path distance (and hence expected morphological distance) between the outer two fossils is always larger than the distance that either of them has from the fossil sampled from an intermediate time. But for samples that straddle bifurcations in a tree, it is quite easy to imagine how this intermediacy could fail; for example, if the two outer taxa lie on one branch of the tree and the fossil from the intermediate time lies on another branch far away (*cf*. Fig. 2). But this example might be unlikely to occur, and indeed we will see that if sampling is uniform across the tree at any given time, in expectation the morphological distances remain intermediate even for this case (*cf*. Fig. 2). Yet for more complex trees, this expected outcome can fail, and perhaps most surprisingly, the distance between the earliest and latest sample can, in expectation, be the *smallest* of the three distances in certain extreme cases.

Thus, in order to make general statements, we will consider the expected degree of relatedness of fossils sampled randomly from given times. Our results will depend solely on the tree shape (including branch lengths) of the underlying tree and the chosen times.

## Results

**Figure 3:**
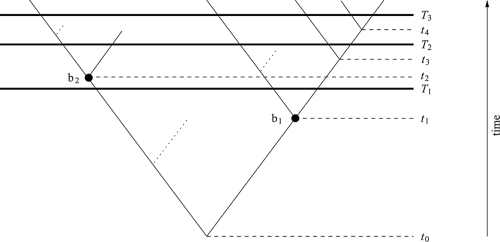
A rooted binary phylogenetic tree with three times *T*_1_, *T*_2_, *T*_3_ at which taxa have been sampled. The dotted branches refer to taxa that do not contribute to the expected distances from one of these times to another and thus are not taken into account. On the other hand, bifurcation *b*_2_ at time *t*_2_ shows that extinction may have an impact on the expected values. Such branches have to be considered.

We begin with some notation. Throughout this paper, we assume a rooted binary phylogenetic tree to be given with an associated time scale 0 < *T*_1_ < *T*_2_ < *T*_3_. The number of *T_i_*-lineages (of lineages extant at time *T_i_*) is denoted by *n_i_*. For instance, in Figure 3, the number *n*_1_ of *T*_1_-lineages is 3, whereas the numbers *n*_2_ and *n*_3_ of *T*_2_- and *T*_3_-lineages are both 5. If not stated otherwise, extinction may occur in the tree. Every bifurcation in the tree is denoted by *b_i_*, where *b*_0_ is the root. Note that in a tree without extinction, the total number of bifurcations up to time *T*_3_ (including the root) is *n*_3_ − 1. For every *b_i_* let *t_i_* denote the time of the occurrence of bifurcation *b_i_*. We may assume that the root is at time *t*_0_ = 0.

Now, for every *b_i_*, we make the following definitions:

$$P_{i}^{j,k}:=n_{j,i}^{l}\cdot n_{k,i}^{r}+n_{j,i}^{r}\cdot n_{k,i}^{l}$$

for all

j,k∈{1,2,3},j≠k

where *n_j,i_^l^* denotes the number of descendants the subtree with root *b_i_* has at time *T_j_* to the left of its root *b_i_*, and *n_j i_^r^* is defined analogously for the descendants on the right hand side of *b_i_*.

It can be seen that bifurcations for which at least one branch of offspring dies out in the same interval where the bifurcation lies always have *P_i_* *^j^*^,^*^k^* -value 0. Consequently, if either *t*_0_ < *t_i_* < *T*_1_ or *T*_1_ < *t*_i_ < *T*_2_ or *T*_2_ < *t_i_* < *T*_3_ and one of *b_i_*’s branches becomes extinct in the same interval, respectively, then *P_i_^j^*^,^*^k^* is 0 for all *j, k*. Note that the number *P_i_^j^*^,^*^k^* denotes the number of different paths in the tree from time *T_j_* to time *T_k_* in the subtree with root *b_i_* and in which no edge is taken twice.

### Example 2.1

*Consider the tree given in *Figure 3. Here, the values P*_i_^j,k^* *for bifurcation b*_1_ *corresponding to time t*_1_ *are P*_1_^1,2^ = *n*_1,1_*^l^* · *n*_2,1_*^r^* + *n*_1,1_*^r^* · *n*_2,1_*^l^* = 1 · 2 + 1 · 1 = 3, *P*_1_^1,3^ = 1 · 3 + 1 · 1 = 4 *and P*_1_^2,3^ = 1 · 3 + 2 · 1 = 5.

In the sampling, select uniformly at random one of the *T_i_*-lineages as well as one of the *T_j_*-lineages to get the expected length *E_i,j_* of the path connecting a lineage at time *T_i_* with one at time *T_j_* in the underlying phylogenetic tree. Then, the expectation that a fossil from the intermediate time *T*_2_ also will be an intermediate taxon of two taxa taken from *T*_1_ and *T*_3_, respectively, refers to the assumption that *E*_1,3_ > max{*E*_1,2_, *E*_2,3_}. We will show in the following lemma that this last inequality can fail and describe the precise condition for this to occur. Moreover, we later show that *E*_1,3_ can be strictly smaller (!) than both *E*_1,2_ and *E*_2,3_—that is the temporally most distant samples can, on average, be more similar than the temporally intermediate sample is to either of the two.

Note that if *P_i_^j^*^,^*^k^* is 0, the corresponding branch does not contribute to the expected distance from one time to another. We can therefore assume without loss of generality that all bifurcations *b_i_* have at least one descendant on their left-hand side and at least one on their right-hand side, each in at least one of the times *T*_1_, *T*_2_, *T*_3_. In Figure 3, branches that therefore need not be considered are represented with dotted lines.

In order to simplify the statement of our results, for all bifurcations *b_i_* set

$$Q_{i}^{j,k}:=\frac{2\cdot P_{i}^{j,k}}{n_{j}n_{k}}$$

for all

j,k∈{1,2,3},j≠k

### Lemma 2.2

*Given a rooted binary phylogenetic tree with times* 0 < *T*_1_ < *T*_2_ < *T*_3_ *and the root at time t*_0_ = 0. Then, *E*_1,3_ ≤ *E*_1,2_ *if and only if*

$$T_{3}-T_{2}\leq \sum_{i:0<t_{i}<T_{1}}(Q_{i}^{1,3}-Q_{i}^{1,2})t_{i}$$

#### Proof

(1) $$\begin{array}{l} E_{1,3}=\frac{1}{n_{1}n_{3}}(\underset{\begin{array}{l} \text{every }T_{3}-\text{lineage}\\ \text{has an ancestor in }T_{1} \end{array}}{\underset{︸}{n_{3}(T_{3}-T_{1})}}\\ +\sum_{i:0<t_{i}<T_{1}}\left[P_{i}^{1,3}(T_{3}-T_{1}+2(T_{1}-t_{i}))\right]\\ +\underset{\text{ways along the root}}{\underset{︸}{P_{0}^{1,3}(T_{3}+T_{1})}}) \end{array}$$

In the above bracket, the three summands refer to different paths from time *T*_1_ to time *T*_3_. The first summand belongs to those paths that go directly from *T*_1_ to *T*_3_ and thus have length *T*_3_−*T*_1_. There are *n*_3_ such ways as every *T*_3_-lineage has an ancestor in *T*_1_. The second summand sums up all paths going along one of the bifurcations *b_i_* for *i* ≠ 0. For every *i*, there are by definition exactly *P_i_*^1,3^ such paths. Similarly, the third summand refers to all paths along the root *b*_0_, whose length is determined by taking the distance from *T*_1_ to the root plus the distance from there to *T*_3_.

As there are altogether *n*_1_*n*_3_ different paths from *T*_1_ to *T*_3_ in the tree, we have:

(2) $$n_{3}+\sum_{i:0<t_{i}<T_{1}}P_{i}^{1,3}+P_{0}^{1,3}=n_{1}n_{3}.$$

Then, by (1) and (2), we get

$$\begin{array}{l} E_{1,3}=\frac{1}{n_{1}}\cdot \frac{1}{n_{3}}\cdot (n_{1}n_{3}T_{3}+(n_{1}n_{3}-2n_{3})T_{1}\\ -2\cdot \sum_{i:0<t_{i}<T_{1}}P_{i}^{1,3}t_{i}), \end{array}$$

and thus

(3) $$E_{1,3}=T_{3}+\frac{n_{1}-2}{n_{1}}T_{1}-\sum_{i:0<t_{i}<T_{1}}Q_{i}^{1,3}t_{i}.$$

Analogously,

(4) $$E_{1,2}=T_{2}+\frac{n_{1}-2}{n_{1}}T_{1}-\sum_{i:0<t_{i}<T_{1}}Q_{i}^{1,2}t_{i}.$$

Thus, with (3) and (4), we can conclude:

$$\begin{array}{l} E_{1,3}\leq E_{1,2}\Leftrightarrow T_{3}-\sum_{i:0<t_{i}<T_{1}}Q_{i}^{1,3}t_{i}\leq T_{2}-\sum_{i:0<t_{i}<T_{1}}Q_{i}^{1,2}t_{i}\\ \Leftrightarrow \;\;\;T_{3}-T_{2}\;\;\;\leq \sum_{i:0<t_{i}<T_{1}}(Q_{i}^{1,3}-Q_{i}^{1,2})t_{i}. \end{array}$$

### Corollary 2.3

*For a given tree there exist times* 0 < *T*_1_ < *T*_2_ < *T*_3_ such that *E_1,3_* ≤ *E_1,2_* if and only if $\sum_{i:0<t_{i}<T_{1}}(Q_{i}^{1,3}-Q_{i}^{1,2})t_{i}>0$.

#### Proof

If, $\sum_{i:0<t_{i}<T_{1}}(Q_{i}^{1,3}-Q_{i}^{1,2})t_{i}\leq 0$, then by Lemma 2.2 we need *T*_2_ ≥ *T*_3_ in order to get *E*_1,3_ ≤ *E*_1,2_. Hence, there are no values 0 < *T*_1_ < *T*_2_ < *T*_3_ such that *T*_3_ − *T*_2_ fulfills the required condition, and so *E*_1,3_ > *E*_1,2_ for all choices of *T_i_*. Conversely, suppose $\sum_{i:0<t_{i}<T_{1}}(Q_{i}^{1,3}-Q_{i}^{1,2})t_{i}>0$. Then, select *T*_1_, *T*_2_ with 0 < *T*_1_ < *T*_2_ and set

$$T_{3}:=\frac{1}{2}\cdot \sum_{i:0<t_{i}<T_{1}}(Q_{i}^{1,3}-Q_{i}^{1,2})t_{i}+T_{2}$$

Then, *T*_3_ > *T*_2_ and

$$\begin{array}{l} T_{3}-T_{2}=\frac{1}{2}\cdot \sum_{i:0<t_{i}<T_{1}}(Q_{i}^{1,3}-Q_{i}^{1,2})t_{i}\\ \leq \sum_{i:0<t_{i}<T_{1}}(Q_{i}^{1,3}-Q_{i}^{1,2})t_{i}. \end{array}$$

By Lemma 2.2, this choice of 0 < *T*_1_ < *T*_2_ < *T*_3_ leads to *E*_1,3_ ≤ *E*_1,2_.

### Corollary 2.4

*If either (i) n*_1_ = 2 *or (ii) no extinction occurs in the tree and n*_2_ = *n*_3_, *then E*_1,3_ > *E*_1,2_.

#### Proof

- (i) Note that if *n*_1_ = 2, obviously only one bifurcation, say *b_î_* (for some *î* such that 0 ≤ *t_î_* < *T_1_*), contributes to the number *n*_1_ of lineages at time *T*_1_, all the branches added by additional bifurcations become extinct before *T*_1_. Thus: *P_î_*^1,3^, *P_î_*^1,2^ ≠ 0 and *P_i_*^1,3^, *P_i_*^1,2^ = 0 for all *i* ≠ *î.* Analogously to the proof of Lemma 2.2 we have for *n*_1_ = 2: *n*_1_*n*_3_ = 2*n*_3_ = *n*_3_ + *P_î_*^1,3^ and *n*_1_*n*_2_ = 2*n*_2_ = *n*_2_ + *P_î_*^1,2^. Thus, *n*_2_ =*P_î_*^1,2^ and *n P_î_*^1,3^. Therefore, *Q_î_*^1,2^ = *Q_î_*^1,3^ 2/*n*_1_ and *Q_î_*^1,2^ = *Q_î_*^1,3^ 0 for all *i* ≠ *î.* Thus, $\sum_{i:0<t_{i}<T_{1}}(Q_{i}^{1,3}-Q_{i}^{1,2})t_{i}=0$ and it follows with Corollary 2.3 that *E*_1,3_ > *E*_1,2_.
- (ii) In this case, obviously *Q_i_*^1,2^ = *Q_i_*^1,3^ for all *i* : 0 < *t_i_* < *T*_1_ and therefore $\sum_{i:0<t_{i}<T_{1}}(Q_{i}^{1,3}-Q_{i}^{1,2})t_{i}=0$. Thus, by Corollary 2.3, *E*_1,3_ > *E*_1,2_.

Lemma 2.2 essentially states that the expected degree of relatedness from taxa of time *T*_1_ to taxa of time *T*_3_ can be larger than the one to taxa of time *T*_2_, but it requires the distance from *T*_2_ to *T*_3_ to be “small enough”. Whether such a solution is feasible can be checked via Corollary 2.3. Lemma 2.2 shows already how the role of intermediates depends on the times the fossils are taken from. Corollary 2.4(i) on the other hand shows how the tree itself has an impact on the expected values: if the tree shape (including branch lengths) is such that at time *T*_1_ only two taxa exist, then the just mentioned scenario cannot happen as the condition of Corollary 2.3 is not fulfilled.

However, we can prove an even stronger result, namely that not only *E*_1,3_ < *E*_1,2_ is possible, but *E*_1,3_ < min{*E*_1,2_, *E*_2,3_} can be obtained for a suitable choice of times *T*_1_, *T*_2_, *T*_3_. For this, we need the following lemma.

### Lemma 2.5

*Given a rooted binary phylogenetic tree with times* 0 < *T*_1_ < *T*_2_ < *T*_3_ *and the root at time t_0_* = 0. *Then E*_1,3_ ≤ *E*_2,3_ *if and only if*

$$\begin{array}{l} \frac{n_{2}-2}{n_{2}}T_{2}-\frac{n_{1}-2}{n_{1}}T_{1}\geq \sum_{i:0<t_{i}<T_{1}}(Q_{i}^{2,3}-Q_{i}^{1,3})t_{i}\\ +\sum_{i:T_{1}<t_{i}<T_{2}}Q_{i}^{2,3}t_{i} \end{array}$$

#### Proof

As in the proof of Lemma 2.2, we have (*cf*. (3))

(5) $$E_{1,3}=T_{3}+\frac{n_{1}-2}{n_{1}}T_{1}-\sum_{i:0<t_{i}<T_{1}}Q_{i}^{1,3}t_{i}.$$

Analogously,

(6) $$E_{2,3}=T_{3}+\frac{n_{2}-2}{n_{2}}T_{2}-\sum_{i:0<t_{i}<T_{2}}Q_{i}^{2,3}t_{i}.$$

Thus, *E*_1,3_ ≤ *E*_2,3_ if and only if

$$\frac{n_{1}-2}{n_{1}}T_{1}-\sum_{i:0<t_{i}<T_{1}}Q_{i}^{1,3}t_{i}\leq \frac{n_{2}-2}{n_{2}}T_{2}-\sum_{i:0<t_{i}<T_{2}}Q_{i}^{2,3}t_{i},$$

which holds precisely if

$$\begin{array}{l} \frac{n_{2}-2}{n_{2}}T_{2}-\frac{n_{1}-2}{n_{1}}T_{1}\geq \sum_{i:0<t_{i}<T_{1}}(Q_{i}^{2,3}-Q_{i}^{1,3})t_{i}\\ +\sum_{i:T_{1}<t_{i}<T_{2}}Q_{i}^{2,3}t_{i}. \end{array}$$

With the help of the two lemmas we can now state the following theorem.

### Theorem 2.6

*Given a rooted binary phylogenetic tree with times* 0 < *T*_1_ < *T*_2_ < *T*_3_ *and the root at time* 0. *Then, E*_1,3_ ≤ min{*E*_1,2_, *E*_2,3_} *if and only if the following two conditions hold*:

$$(i)\;T_{3}-T_{2}\leq \sum_{i:0<t_{i}<T_{1}}(Q_{i}^{1,3}-Q_{i}^{1,2})t_{i},$$

$$\begin{array}{l} (ii)\;\frac{n_{2}-2}{n_{2}}T_{2}-\frac{n_{1}-2}{n_{1}}T_{1}\geq \sum_{i:0<t_{i}<T_{1}}(Q_{i}^{2,3}-Q_{i}^{1,3})t_{i}\\ +\sum_{i:T_{1}<t_{i}<T_{2}}Q_{i}^{2,3}t_{i}. \end{array}$$

#### Proof

The Theorem follows directly from Lemmas 2.2 and 2.5.

The following example demonstrates the influence of times 0 < *T*_1_ < *T*_2_ < *T*_3_ according to the above theorem.

### Example 2.7

Consider again Figure 3.

1. *Assume t*_1_ = 15, *T*_1_ = 100, *t*_2_ = 107, *t*_3_ = 109, *T*_2_ = 110, *T*_3_ = 130. *Then*, *E*_1,2_ = 137.33, *E*_2,3_ = 155.28 *and E*_1,3_ = 155.33. *Hence, for this choice of times, we have E*_1,3_ > max{*E*_1,2_, *E*_2,3_}.
2. *Consider the same times as in the previous case, but choose T*_2_ = 129 *instead of T*_2_ **=** 110. *This means to move T*_2_ *further away from T*_1_ *and closer to T*_3_. *This change is enough to give completely different expected values: E*_1,2_ = 156.33, *E*_2,3_ = 166.68 and *E*_1,3_ = 155.33. *Hence, for this choice of times, we have E*_1,3_ < min{E_1,2_, E_2,3_}.

## Discussion

The analysis of the fossil record provides an insight into the history of species and thus into evolutionary processes. Stochastic models can provide a useful way to infer patterns of diversification, and they form a useful link between molecular phylogenetics and paleontology [8]. Such models would greatly benefit from incorporation of potential fossil ancestors and other extinct data points to infer patterns of evolution. In this paper we have applied a simple model-based phylogenetic approach to study the expected degree of similarity between fossil taxa sampled at intermediate times.

‘Gaps’ in the fossil record are problematic [10] as they can be interpreted as ‘missing links’. Therefore, numerous studies concerning the adequacy of the fossil record have been conducted (see, for example, [3], [9], [13]), and it is frequently found that even the available fossil record is still incompletely understood. This is particularly true for ancestor-descendant relationships (see, for instance, [4], [5]). For example Foote [5] reported the probability that a preserved and recorded species has at least one descendant species that is also preserved and recorded is on the order of 1%–10%. This number is much higher than the number of identified ancestor-descendant pairs. Thus, it remains an important challenge to recognize such pairs [1]. This is also essential with regard to ancestor-intermediate-descendant triplets, as it is possible that there are in fact fewer ‘gaps’ than currently assumed, i.e. that intermediates are present but not yet recognized. Such issues have an important bearing on any conclusions our results might imply concerning the testing of hypotheses of continuous morphological evolution, or concerning the shape of the underlying evolutionary tree based on the non-existence of certain intermediates.

Another challenge is to investigate different phylogenetic models for describing the expected degree of morphological separation between different fossil taxa sampled at different times. Our findings strongly depend on the assumption that morphological diversification is proportional to the distance in the underlying phylogenetic tree. This is justified if morphological difference is proportional to the number of differing discrete characters, that each of these characters changes at a constant rate over the time period of sampling, and that homoplasy is rare. This last assumption requires the rate of character change to be sufficiently small in relation to the time period of the sampling—the appearance of reverse or convergent character states will lead to a more concave (rather than linear) relationship between morphological divergence and path distance. A similar concave relationship might be expected for continuous morphological evolution as described by neutral Brownian-motion.

Thus, the impact of different assumptions on the role of intermediates could be further investigated. But even if we assume that diversification is proportional to time, there may be other ways to measure ‘distance’ that could be usefully explored—for instance, one could define the distance between two taxa to be the maximum (rather than the sum) of the two divergence times of the taxa back to their most recent common ancestor. This definition of distance allows the degree of relatedness to be higher for taxa on the same clade than for other taxa. In this case, there exist analogous results to Lemmas 2.2 and 2.5 (results not shown), but the formulae are somewhat different, particularly for Lemma 2.5.
